# Addressing the Transition to Adult Health Care for Adolescents and Young Adults with Pancreatic Disorders

**DOI:** 10.7759/cureus.57972

**Published:** 2024-04-10

**Authors:** Laura Hart, Cheryl Gariepy, Jason F Woodward, Luis F Lara, Darwin Conwell, Maisam Abu-El-Haija

**Affiliations:** 1 Internal Medicine/Pediatrics, Nationwide Children’s Hospital, Columbus, USA; 2 Pediatric Gastroenterology, Nationwide Children’s Hospital, Columbus, USA; 3 Adolescent and Transition Medicine, Cincinnati Children's Hospital Medical Center, Cincinnati, USA; 4 Gastroenterology and Hepatology, The Ohio State University College of Medicine, Columbus, USA; 5 Internal Medicine/Gastroenterology, University of Kentucky College of Medicine, Lexington, USA; 6 Pediatric Gastroenterology, Cincinnati Children's Hospital Medical Center, Cincinnati, USA

**Keywords:** young adult health, adolescent health, chronic pancreatitis, recurrent acute pancreatitis, transition to adult health care

## Abstract

Introduction: The transition from pediatric to adult health care is a vulnerable time period for adolescents and young adults (AYA). Guidance on how to effectively implement transition support for AYA with recurrent acute pancreatitis (RAP) and chronic pancreatitis (CP) is lacking.

Methods: To address this gap, we formed a consortium of pancreatic centers that would work in coordination to test interventions to improve the transition for AYA with RAP and CP. We then performed a baseline assessment of consortium resources and patient transition readiness and developed an educational toolkit for AYA with RAP and CP.

Results: Our consortium consists of three National Pancreatic Centers of Excellence, each with a multidisciplinary team to work with AYA with RAP and CP. While our patients ages 18 to 23 were generally seen at the pediatric centers, the baseline assessment of transition readiness suggests that our patients may have higher transition readiness scores than other populations. The educational toolkit contains both pancreas-specific and general guidance to support AYA with RAP and CP during their transition, including guidance on nutrition, pain management, and finding an adult gastroenterologist.

Conclusions: We have formed a consortium to test interventions to improve the transition to adult health care for AYA with RAP and CP. We have completed a baseline assessment and developed our first intervention: an educational tool kit. Future work planned includes tests of the tool kit and efforts to improve rates of transfer to an adult provider for YA with RAP and CP.

## Introduction

Recurrent acute pancreatitis (RAP) and chronic pancreatitis (CP) are diseases that can be identified in childhood and continue to adulthood [1, 2]. RAP & CP negatively impact global health status, physical functioning, and quality of life (QOL) in both children and adults. Most 18-24-year-old young adults (YAs) with childhood-onset RAP and CP getting specialized medical care continue to be managed by pediatric providers, who are not trained in caring for health issues that affect adult bodies that can involve issues like sexuality, family planning, and substance use, and complications of common adult chronic diseases. Thus, it is recommended that YA transition their health care from pediatric to adult providers, who are better-trained for adult health needs [1, 2].

The transition from pediatric to adult health care (P2A) is a shift from pediatric, parent-supervised healthcare to independent, patient-centered healthcare, with or without a transfer to a new provider [3, 4]. It represents a vulnerable period that can result in poor adherence to treatment, disruption in follow-up care, emergent medical complications, and general health deterioration [5-7]. Expert recommendations for addressing the transition for YA with RAP and CP have been published [8]. Both these recommendations and more general guidelines for addressing transition recommend a structured P2A transition program for youth with special healthcare needs [4, 8]. Currently, however, there are no established transition readiness criteria for adolescents and YA (AYA) with RAP and CP and little has been published about strategies for implementing the current transition guidance for this population in clinical care.

To address this gap, we formed a consortium among our pancreatic centers to develop and test interventions to improve the transition to adult health care for AYA with RAP and CP. We have determined our baseline resources and patient volumes, assessed transition readiness among the patients served by the consortium, and developed our initial intervention: an educational toolkit for AYA with RAP and CP.

## Materials and methods

Setting

The consortium consists of three centers, all located in Ohio: the Pancreas Care Center at Cincinnati Children’s Hospital Medical Center (CCHMC), the Nationwide Children’s Hospital (NCH) Pancreas and Liver Care Center, and the Pancreas Clinic at The Ohio State University Wexner Medical Center, the latter of which provides pancreas care for adults. All three centers are designated as National Pancreas Foundation Centers of Excellence. This initiative was started as an ancillary study under the Consortium for the Study of Chronic Pancreatitis, Diabetes, and Pancreatic Cancer (CPDPC).

Baseline assessment of P2A consortium resources and patient volumes

Our baseline assessment consisted of determining the available resources at each institution for addressing transition for YA with RAP and CP as well as determining the number of patients ages 18 to 23 in need of transition support across the three centers. 

Transition readiness assessment

As a preliminary assessment of transition readiness in our patients with RAP and CP, we reviewed the results of the Transition Readiness Assessment Questionnaire (TRAQ) 5.0, which was distributed to patients 15 years to 24 years old who attended clinic appointments in December 2021 through April 2022 at CCHMC. Clinical staff assigned the questionnaire to patients ages 15 to 24 years old with RAP and CP via the patient’s electronic medical record (EMR) during pre-visit planning. This questionnaire was filled out by patients via MyChart or a tablet provided by the clinic staff before the patient’s appointment. Data from the questionnaire was automatically uploaded into the patient’s EMR for the clinician to evaluate during the patient’s clinic visit. We retrospectively assessed these results to understand baseline data regarding transition readiness in this population.

The TRAQ questionnaire has been validated in youth with chronic illnesses [9-11], and it has been used for several studies addressing transition readiness [12-20]. The questionnaire includes twenty questions that can be divided into five subdomains. Each question has a five-level Likert scale response using the following responses: 1) no, I do not know how; 2) no, but I want to learn; 3) no, but I am learning to do this; 4) yes, I have started doing this; 5) yes, I always do this when I need to. For the purposes of this study, overall scores and domain scores have been calculated as a mean of the patient’s answers, as has been done elsewhere [12, 14-16], so that domains and overall scores can be easily compared. The range for the overall score and each domain score is one to five, with higher scores indicating greater levels of transition readiness. 

Education kit development

The educational toolkit was created using input from a review of the transition literature and then edited with guidance from successful transition programs within CCHMC and the input of experts in transitional care. The literature search identified resources from the National Pancreas Foundation (NPF) to promote patient knowledge and self-management strategies including education about the pancreas in general, healthy eating information and recommended recipes, support groups, education about coping with pain, and information on how to choose an NPF Center of Excellence. These pancreas-specific resources were combined with several resources from Got Transition®, a national program of the National Alliance to Advance Adolescent Health funded by the Maternal Child Health Bureau to provide support for the P2A transition for patients, families, and providers [21, 22]. Specifically, we utilized the digital transition readiness quiz, information on setting up a medical ID on a cell phone, and considerations for the first appointment with an adult gastroenterologist from the Got Transition website.

The structure of the educational toolkit was based on the format of the transition toolkit utilized for pediatric patients with inflammatory bowel disease (IBD) at CCHMC [23] so that it would be most useful to patients as they make the transition to adult health care. We structured our toolkit to have a similar set of sections in it, including information about the importance of transitioning to adult care, the timeline for transfer to adult care, an age-based checklist of transition tasks and skills, as well as general adherence tips, healthy diet education, understanding insurance, and self-management problem-solving scenarios. We then used this general structure and included pancreas-specific information and resources to make the toolkit applicable to AYA with RAP and CP. Two pediatric pancreatologists (MAH and CG) and one adult pancreatologist (LL) met regularly (twice a month during the period of creating the content) with the transition experts on the team (JW, LCH) to apply pancreas-specific criteria to the transition methods. Additionally, a draft of the toolkit was reviewed with 3 families to get their feedback and make adjustments to improve the toolkit contents. 

This study was approved (No. 2023-0440) by the CCHMC Institutional Review Board (IRB) with a waiver of consent. 

## Results

Baseline assessment results

Across the three institutions, our team included pediatric pancreatologists, adult pancreatologists, experts in transition, nurses, social workers, and psychologists, allowing for a multi-disciplinary perspective and approach to transition. None of the centers have dedicated transition support in the clinic at this time. Our analysis of the patient counts found that most (69%) of the 472 YA ages 18 to 23 with RAP and CP seen in our consortium were being seen at one of the pediatric centers, 49% at CCHMC, and 20% at NCH. The differences between the pediatric centers are in part due to CCHMC seeing more patients overall (796 at CCHMC vs. 653 at NCH), and in part due to CCHMC seeing a higher proportion of adults compared to NCH (41% at CCHMC vs. 27% at NCH). 

Notably, each center has differing levels of institutional support for transition. For example, CCHMC has the TRAQ embedded in its electronic medical record, so this can be completed by patients and monitored as part of routine clinical care. 

Transition readiness assessment

A total of 38 patients responded to the TRAQ questionnaire during their clinic visits within the Pancreas Care Center at CCHMC between December 2021 and April 2022. Demographic characteristics are shown in Table 1. Scores from the participants are separated by age and TRAQ domain in Table 2. Overall, scores increased with patient age (mean overall score of 3.3 for those of age 15 vs. 4.39 for those of age 18+). Scores tended to vary across domains. Patients in the 15-17-year-old range self-identified managing medications, appointment keeping, and tracking health issues as skills they lacked mastery with. Notably, all age groups reported high scores in the managing daily activities domain (range of 4.37 to 4.78 across age groups).

**Table 1:** Demographic Characteristics of Patients Completing the TRAQ TRAQ: Transition Readiness Assessment Questionnaire * Percents add up to > 100% due to rounding

	N/Mean	%/SD
Total number of patients	38	
Gender (n, %)		
Male	15	39
Age Breakdown (n, %)		
15	11	29
16	9	24
17	9	24
18+	9	24
Race (n, %)		
White	27	71
Black/African American	5	13
Other	5	13
Missing	1	3
Hispanic (n, %)		
Yes	4	11
TRAQ Score (mean, SD)	3.7	0.8

**Table 2:** Mean Scores by Age for the TRAQ Domains and Overall TRAQ Scores TRAQ: Transition Readiness Assessment Questionnaire

Age Group	Mean Scores for the TRAQ Domains					Mean Overall TRAQ score
	Managing Medications	Appointment Keeping	Tracking Health Issues	Talking with Providers	Managing Daily Activities	
15 (n=11)	3.45	2.48	2.85	3.85	4.52	3.3
16 (n=9)	4.15	2.67	2.70	3.96	4.30	3.51
17 (n=9)	4.11	3.33	2.93	4.44	4.44	3.71
18 & up (n=9)	4.53	4.13	4.37	4.48	4.78	4.44

Toolkit components

**Figure 1:**
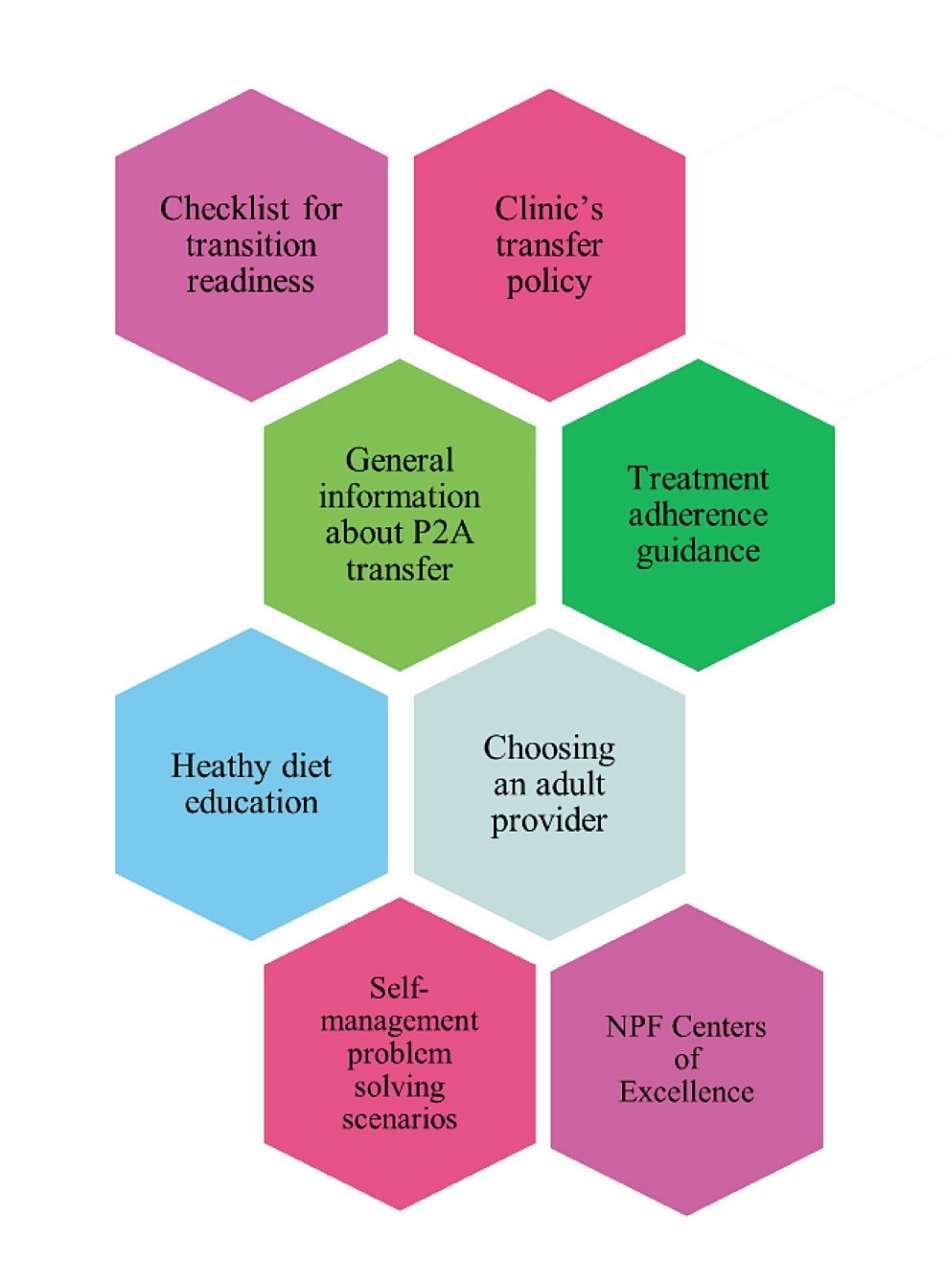
Components of the Pediatric to Adult (P2A) Educational Toolkit

The P2A educational toolkit includes a checklist for transition readiness, the clinic’s transfer policy, information about transitioning to adult care, treatment adherence guidance, healthy diet teaching, self-management problem-solving scenarios, assistance on choosing an adult provider, and details about the National Pancreas Foundation Centers of Excellence (Figure 1). The toolkit will be delivered to patients via paper copy in the clinic to allow for education from providers to occur in the clinic during the visit when the toolkit is provided. See Appendices for sample text from the transition toolkit. Full details are available on request by contacting the authors. 

The educational toolkit is intended to be given to adolescents to reinforce the concept of transitioning care to an adult provider, address any knowledge or skills gaps before leaving pediatric care, and encourage a conversation about transition with the health care team. Information about the importance of transitioning to adult doctors and a general timeline for transfer is included as an introduction to the P2A process.

The checklist included in the toolkit provides guidance on specific tasks of transition for AYA to consider next as they increase in self-management of their care. The checklist uses age as a guide, but AYA who utilize the checklist will not be required to strictly adhere to the age recommendations. The toolkit also explains why adherence is important and provides hints for adherence to appointments, medications, and treatments.

There are QR codes throughout the document that provide links to helpful websites regarding support groups, NPF centers of excellence, setting up a medical ID in your phone, and coping with pain. There are problem-solving scenarios to help the teenager critically think about self-management. The toolkit concludes with guidance on choosing an adult provider, including factors to consider when choosing an adult provider and strategies for finding an adult provider, determining if the adult provider takes your insurance, and questions to guide the YA’s first appointment with an adult provider.

## Discussion

In this work, we established a P2A consortium to address the transition from pediatric to adult health care for AYA with RAP and CP. As an initial step toward our larger goals, we ascertained the available resources within the consortium for addressing transition-related care, completed a preliminary assessment of the transition readiness of patients, and developed an educational toolkit to support adolescents with RAP and CP as they transition to adult health care.

We found that most YA with RAP and CP were being seen at the pediatric centers in the consortium, suggesting that improved procedures for transferring patients from pediatric to adult health care are needed and that the consortium has the numbers to serve as a learning laboratory for improving the transition and transfer process for YA with RAP and CP. We also found areas where AYA reported needing assistance, such as tracking health issues. The overall means for the age groups are similar to the 75th percentiles for transition readiness scores among adolescents and young adults with IBD [16], suggesting that the group assessed for the baseline assessment may have higher levels of readiness than other groups, though we will need to expand our assessment across centers to confirm this in a larger diverse sample. The fact that most YA continues to be seen in the pediatric health care setting suggests the need to explore the possibility of transfer barriers beyond individual independence in health care management in pancreatic disease, which is consistent with other work that showed that transition readiness did not correlate with a transfer to adult health care [24]. In our collaboration, we found that youth’s self-reported transition readiness increases with age, consistent with previous work [10, 25-28]. 

The educational toolkit is the first pancreas-specific resource for the P2A transition. After changes are tested and then implemented on a small scale within CCHMC and NCH, the P2A education kit will be spread to several pancreas centers. Although most components of the P2A packet are disease-specific, it is not organization-specific. It is intended to be implemented in various settings caring for pediatric patients with pancreatic disorders. Many YA with chronic conditions, such as IBD and diabetes, have requested support regarding the topics addressed in the toolkit, and so we feel this addresses an important knowledge gap for YA with RAP and CP [29, 30]. 

As we make the next steps to implement the educational toolkit in clinical practice, we plan to continue to track transition readiness levels to assess what information patients and families feel they have mastered and where they feel like more support is needed. This includes expanding the use of transition readiness assessment tools to the other institutions in the consortium. Additionally, the toolkit itself has checks on patient knowledge and problem-solving, allowing for more objective assessments of transition-related knowledge and skills to be completed in the clinic. 

The formation of the consortium and a common set of tools to support transition also provide an opportunity for future work to understand how different approaches to transition affect the experience of YA with RAP and CP as they move to adult gastroenterology care. We hope to engage in such work in the future.

This study is limited in that the readiness data was collected at one pediatric center, so baseline data may not generalize to other sites. We utilized the TRAQ 5.0, which had some challenges when put to practical use that have since been addressed by the development of the TRAQ 6.0 [11]. If future work makes use of TRAQ 6.0, it will be difficult to compare to the data found here. We did not seek specific feedback from adolescents or young adults during the development of the toolkit, but intend to do so during the implementation phase.

## Conclusions

In conclusion, we developed an educational toolkit to provide information and support to adolescents and young adults with recurrent acute pancreatitis and chronic pancreatitis and their families during the transition to adult healthcare. We then assessed the transition readiness of youth in our center to determine the next steps for deploying the toolkit in the clinic. Future work will include utilizing the toolkit in the clinic with patients and families and refining the materials while tracking the outcomes from the pediatric to adult health care transition and its impact on disease progression. This will allow broader dissemination to other pediatric to adult pancreas centers and wider adoption in the future.

## Appendices

Sample text from the transition toolkit

Why Transition?

As a teenager, you aren’t a child anymore! You are on the road to becoming an independent adult. As an adult with pancreatic disorders, it is important for you to understand your health and be able to manage it independently. You will need support from family, friends, and your pancreas care team. We want to help prepare you for life as an adult with pancreatic disorders so that when the time comes, you’ll be ready to transfer your care to adult pancreas doctors. Adult pancreas providers are experts at dealing with adult issues and managing pancreas care. You will be most successful if you are prepared at the time of transfer of care. We hope that our transition program will get you ready!

Timeline for Transfer to Adult Pancreas Care

While we will be discussing preparation for transfer to adult pancreas providers during clinic visits, we want to reassure you that transfer to adult care happens at different times for each patient. There is no set age when a patient must transfer. The discussion on decision and timeline for transfer is based on many things, such as medical stability, plans for college or employment, insurance coverage, etc.

When the time does come to change from your pediatric pancreas doctors to adult pancreas doctors, we are going to assist you through this step-by-step process. This includes: 1) providing you with options to choose from for your new adult pancreas doctors, 2) Ensuring the new doctor accepts your insurance, 3) discussing office location(s) for the new doctor, and 4) sending your pediatric medical records to the new doctor’s office.
